# Supplementary material for: Content-rich biological network constructed by mining PubMed abstracts
Source: BMC Bioinformatics. 2004 Oct 8;5:147. doi: 10.1186/1471-2105-5-147 (PMC528731; doi:10.1186/1471-2105-5-147)
Supplement: Additional File 2 — The original results of the above study (non-essential files are deleted to keep the file size under the limit set by BMC bioinformatics). [file 1471-2105-5-147-S2.bz2 › chilibotAdditionalFile2/dip10/108ID7559507E445/html/IGF1R_IRS1.html]

 


 **IGF1R** and **IRS1** 
  
Found 109 abstracts in PubMed, retrieved 10.  
 

 What does Google say? 
 PDF only 
| .edu only 

---

- J NutrJ Nutr,   **Conjugated Linoleic Acid Downregulates Insulin Like Growth Factor I Receptor **[IGF1R]** Levels in HT 29 Human Colon Cancer Cells.** .
  Conjugated linoleic acid CLA has chemoprotective properties in a variety of experimental cancer models.
  We have previously observed that dietary CLA inhibits colon tumorigenesis induced by dimethylhydrazine in rats.
  In addition, our in vitro studies have shown that CLA inhibits DNA synthesis and induces apoptosis in HT 29 cells, the human colon adenocarcinoma cell line.
  The insulin like growth factor IGF system regulates the growth of HT 29 cells by an autocrine mechanism.
  The present study examined whether the growth inhibitory effect of CLA is related to changes in the IGF system in HT 29 cells.
  To determine whether CLA inhibits IGF II production, HT 29 cells were incubated in serum free medium in the presence of various concentrations of CLA.
  CLA decreased protein levels of both mature and pro IGF II and IGF II transcripts.
  Whereas exogenous IGF I and IGF II produced an increase in cell number, neither IGF I nor IGF II counteracted the negative growth regulatory effect of CLA.
  Reverse transcriptase polymerase chain reaction and Western blot analysis of total cell lysates revealed that CLA decreased IGF I receptor IGF IR transcript and protein levels in a dose dependent manner.
  Immunoprecipitation Western blot studies revealed that CLA inhibited IGF I induced phosphorylation of IGF IR and insulin receptor substrate IRS 1 **[IRS1]**, recruitment of the p85 regulatory subunit of phosphatidylinositol 3 kinase PI3K to IGF IR, IGF IR associated PI3K activity, and phosphorylated Akt and extracellular signal regulated kinase ERK 1 2 levels.
  In conclusion, the inhibition of cell proliferation and induction of apoptosis by CLA in HT 29 cells may be mediated in part by its ability to decrease IGF II synthesis and to downregulate IGF IR signaling and the PI3K Akt and ERK 1 2 pathways.

  - Endocrinology< MedlineTA>Endocrinology,   **Cytokine Hormone Interactions Tumor Necrosis Factor alpha Impairs Biologic Activity and Downstream Activation Signals of the Insulin Like Growth Factor I Receptor **[IGF1R]** in Myoblasts.** .
    TNFalpha is elevated following damage to skeletal muscle.
    Here we provide evidence that TNFalpha acts on muscle cells to induce a state of IGF I receptor resistance.
    We establish that TNFalpha inhibits IGF I stimulated protein synthesis in primary porcine myoblasts.
    Similar results were observed in C 2 C 12 murine myoblasts, where as little as 0.01 ng ml TNFalpha significantly inhibits protein synthesis induced by IGF I.
    TNFalpha also impairs the ability of IGF I to induce expression of a key myogenic transcription factor, myogenin.
    The inhibition by TNFalpha of IGF I induced protein synthesis and expression of myogenin is not due to direct killing of myoblasts by TNFalpha.
    Although IGF I induces an approximately 19 fold induction in tyrosine phosphorylation of the beta chains of its receptor, TNFalpha does not inhibit this autophosphorylation.
    Instead, TNFalpha significantly reduces by approximately 50% IGF I stimulated tyrosine phosphorylation of two of the major downstream receptor docking molecules, insulin receptor substrate IRS 1 **[IRS1]** and IRS **[IRS1]** 2.
    These results establish that low picogram concentrations of TNFalpha acts on both porcine and murine myoblasts to impair tyrosine phosphorylation of both IRS 1 **[IRS1]** and IRS **[IRS1]** 2, but not the receptor itself.
    These data are consistent with the notion that very low physiological concentrations of TNFalpha interfere with both protein synthesis and muscle cell development by inducing a state of IGF I receptor resistance.

    - Cancer Res, 2003   Insulin like growth factors stimulate breast cancer proliferation, motility, and survival.The type I IGF receptor mediates the effects of IGF I.
      Thus, inhibition of **IGF1R** activation could inhibit IGF action in breast cancer cells.
      A single chain antibody directed against **IGF1R** has been shown to partially inhibit xenograft growth of MCF 7 cells in athymic mice.
      In this study, we have examined the effects of scFv Fc on **IGF1R** signaling in the estrogen receptor positive MCF 7 breast cancer cells in vitro and in vivo.
      The antibody stimulated **IGF1R** activation in vitro in MCF 7 cells and was unable to block IGF I effects.
      The antibody also stimulated proliferation of MCF 7 cells in monolayer growth assays.
      To determine how scFv Fc could stimulate in vitro growth yet inhibit in vivo tumor growth, we examined the effect of scFv Fc on **IGF1R** expression.
      In MCF 7 cells, scFv Fc down regulated **IGF1R** levels after 2 h, and the levels were greatly reduced after 24 h.
      In contrast, IGF I treatment over the same time period did not affect **IGF1R** levels.
      Twenty four h pretreatment of cells with scFv Fc blocked IGF I mediated phosphorylation of insulin receptor substrate 1 **[IRS1]** and subsequent extracellular signal regulated kinase 1 extracellular signal regulated kinase 2 and phosphatidylinositol 3 kinase activation.
      In contrast, cells treated with 5 nM IGF I for 24 h still retained the ability to further activate downstream signaling pathways in response to IGF I.
      Moreover, pretreatment of MCF 7 cells with scFv Fc rendered them refractory to further proliferation induced by additional IGF I.
      Twenty four h pretreatment of cells with scFv Fc also inhibited IGF I stimulated anchorage independent growth.
      scFv Fc did not enhance antibody dependent cell mediated cytotoxicity.
      In vivo, treatment of mice bearing MCF 7 xenograft tumors with scFv Fc resulted in near complete down regulation of **IGF1R**.
      Our data show that scFv Fc stimulates biochemical activation of **IGF1R**, then causes receptor down regulation, making MCF 7 cells refractory to additional IGF I exposure.
      These results indicate that such chimeric single chain antibodies against **IGF1R** have future potential in breast cancer therapy by causing down regulation of receptor.

      - Oncogene, 2003   **An inhibitory function for JNK in the regulation of IGF I signaling in breast cancer.** .
        Insulin like growth factor I receptor **[IGF1R]** IGF IR is frequently overexpressed in a variety of cancer types.
        Since many breast tumors and cancer cell lines overexpress IGF IR, we tested IGF I effects on chemotherapy treated breast cancer cells.
        IGF I protects from chemotherapy induced apoptosis, suggesting that overlapping signaling pathways modulate IGF I and chemotherapy treatment outcomes.
        Taxol and other chemotherapy drugs induce c Jun N terminal kinase JNK , a kinase that conveys cellular stress and death signals.
        Notably, in this paper we show that IGF I alone induces a potent JNK response and this activity is reversed by inhibition of phosphatidylinositol 3 kinase PI 3 kinase with LY294002 in MCF 7 but not T47D cells.
        Cotreatment of cells with chemotherapy and IGF I leads to additive JNK responses.
        Using cells overexpressing Akt, we confirm that IGF I mediated survival is Akt dependent.
        In contrast, overexpression of JNK significantly enhances Taxol induced apoptosis and inhibits IGF I survival effects.
        Further, JNK attenuates anchorage independent growth of MCF 7 cells.
        The inhibitory effect of JNK appears to be mediated by serine phosphorylation of IRS 1 **[IRS1]** insulin receptor substrate since both Taxol and IGF I treatment enhanced Ser 312 IRS 1 **[IRS1]** phosphorylation, while LY294002 blocked IGF I mediated phosphorylation.
        Taken together, these data provide a mechanism whereby stress or growth factors activate JNK to reduce proliferation and or survival in breast cancer cells.

        - Mol Endocrinol< MedlineTA>Mol Endocrinol,   The insulin receptor substrate 1 **[IRS1]** can translocate to the nuclei and nucleoli of several types of cells.
          Nuclear translocation can be induced by an activated insulin like growth factor 1 receptor **[IGF1R]** , and by certain oncogenes, like the SV40 T antigen and v src.
          We have asked whether IRS **[IRS1]** 2 could also translocate to the nuclei.
          In addition, we have studied the effects of functional mutations in the IGF IR on nuclear translocation of IRS **[IRS1]** proteins.
          IRS **[IRS1]** 2 translocates to the nuclei of mouse embryo fibroblasts expressing the IGF IR, but, at variance with IRS 1 **[IRS1]**, does not translocate in cells expressing the SV40 T antigen.
          Mutations in the tyrosine kinase domain of the IGF IR abrogate translocation of the IRS **[IRS1]** proteins.
          Other mutations in the IGF IR, that do not interfere with its mitogenicity but inhibit its transforming capacity, result in a decrease in translocation, especially to the nucleoli.
          Nuclear IRS 1 **[IRS1]** and IRS **[IRS1]** 2 interact with the Upstream Binding Factor , which is a key regulator of RNA polymerase I activity and, therefore, ribosomal RNA synthesis.
          In 32D cells, wild type, but not mutant IRS 1 **[IRS1]**, causes a significant activation of the rDNA promoter.
          The interaction of nuclear IRS **[IRS1]** proteins with UBF1 constitutes the first direct link of these proteins with the rDNA transcription machinery.

          - Endocrinology, 2003   **Essential role of insulin like growth factor I receptor **[IGF1R]** in insulin induced fetal brown adipocyte differentiation.** .
            To define the specific role of IGF I receptor IGF IR in adipogenic and thermogenic differentiation of brown adipocytes during late fetal life, we have established immortalized brown adipocyte cell lines from fetuses of IGF IR deficient mice IGF IR as well as from wild type mice IGF IR .
            IGF IR cells showed an increased insulin sensitivity regarding insulin receptor substrate 1 **[IRS1]** IRS 1 **[IRS1]** tyrosine phosphorylation despite a substantial reduction in IRS 1 **[IRS1]** protein content.
            Furthermore, insulin induced total and IRS 1 **[IRS1]** associated phosphatidylinositol 3 kinase activities were augmented in IGF IR deficient cells compared with wild type cells.
            Downstream phosphatidylinositol 3 kinase activation of Akt, but not p70s6 kinase, were elicited at lower doses of insulin in IGF IR brown adipocytes.
            Activation of protein kinase Czeta by insulin was similar in both cell types as was insulin induced glucose uptake.
            Treatment of wild type brown adipocytes with insulin for 12 h up regulated fatty acid synthase FAS and adipocyte determination and differentiation ADD1 SREBP mRNAs.
            this effect was impaired in the absence of IGF IR.
            At the protein level, insulin increased FAS content and the amount of the mature form of adipocyte determination and differentiation ADD1 SREBP in the nucleus in wild type cells, but not in IGF IR cells.
            Furthermore, 24 h of insulin stimulation induced the expression of both uncoupling protein 1 and CCAAT enhancer binding protein alpha C EBPalpha in wild type brown adipocytes.
            these effects were abolished in IGF I R cells.
            Retrovirus mediated reexpression of peroxisomal proliferator activated receptor gamma PPARgamma in IGF IR brown adipocytes could overcome FAS mRNA impairment, bypassing insulin signaling.
            However, insulin further increased FAS mRNA expression in C EBPalpha IGF IR cells, but not in PPARgamma IGF IR cells.
            In addition, fetal brown adipocytes lacking IGF IR up regulated uncoupling protein 1 expression in the absence of insulin when PPARgamma, but not C EBPalpha, was overexpressed.
            These data provide strong evidence for a critical role of IGF IR in the differentiation of the brown adipocyte phenotype in fetal life.
            this effect is mimicked by PPARgamma in an insulin independent manner.

            - J Radiat Res Tokyo , 2002   **The phosphatidylinositol 3 kinase pathway is not essential for insulin like growth factor I receptor **[IGF1R]** mediated clonogenic radioresistance.** .
              The insulin like growth factor I receptor **[IGF1R]** IGF IR is known to induce clonogenic radioresistance in cells following ionizing irradiation.
              To explore the downstream signaling pathways, we focused on the phosphatidylinositol 3 kinase PI3 K pathway, which is thought to be the primary cell survival signal originating from the receptor.
              For this purpose, R cells deficient in the endogenous IGF IR were used as a recipient of the human IGF IR with or without mutations at potential PI3 K activation sites NPXY950 and Y1316XXM.
              Mutants with double mutation at Y950 Y1316 exhibited not abrogated, but reduced activation of IRS 1 **[IRS1]**, PI3 K, and Akt upon IGF I stimulation.
              However, the mutants had the same clonogenic radioresistance as cells with wild type WT receptors.
              Neither wortmannin nor LY294002, specific inhibitors of PI3 K, affected the radioresistance of cells with WT receptors at concentrations specific for PI3 K.
              Collectively, these results indicate that the PI3 K pathway is not essential for IGF IR mediated clonogenic radioresistance.

              - J Biol Chem, 2003   **Redundancy of radioresistant signaling pathways originating from insulin like growth factor I receptor **[IGF1R]**.** .
                The insulin like growth factor I receptor **[IGF1R]** IGF IR has the ability to confer clonogenic radioresistance following ionizing irradiation.
                We attempted to determine the downstream pathways involved in IGF IR mediated radioresistance and used mouse embryo fibroblasts deficient in endogenous IGF IR R as recipients for a number of mutant IGF IRs **[IRS1]**.
                Mutational analysis revealed that the tyrosine at residue 950 Tyr 950 of IGF IR, as well as the C terminal domain, are required for radioresistance.
                both domains must be mutated to abrogate the phenotype.
                Furthermore, the contribution of downstream pathways was analyzed by combining the use of wild type or Tyr 950 and C terminal mutants with specific inhibitors of phosphatidylinositol 3 kinase PI3 K or mitogen activated protein extracellular signal regulated kinase ERK kinase MEK .
                Radioresistance could be induced by IGF IR as long as the ability of the receptor to stimulate the MEK ERK pathway was retained.
                This was confirmed by the expression of constitutively active MEK in R cells.
                The ability to stimulate the PI3 K pathway alone was not sufficient, but PI3 K activation coupled with MEK ERK pathway independent signals from the C terminus was able to induce radioresistance.
                Taken together, these results indicate that the IGF IR mediated radioresistant signaling mechanism progresses through redundant downstream pathways.

                - J Neurovirol, 2002   **Insulin like growth factor I receptor **[IGF1R]** signaling system in JC virus T antigen induced primitive neuroectodermal tumors medulloblastomas.** .
                  Medulloblastomas represent about 25% of all pediatric intracranial neoplasms.
                  These highly malignant tumors arise from the cerebellum, affecting mainly children between ages 5 and 15.
                  Although the etiology of medulloblastomas has not yet been elucidated, several reports suggest that both the cellular protein insulin like growth factor I IGF I and the early protein of the human polyomavirus JC JCV T antigen may contribute to the development of these tumors.
                  The results of this study show a potential functional cooperation between these two proteins in the process of malignant transformation.
                  Both medulloblastoma cell lines and medulloblastoma biopsies are characterized by the abundant presence of the IGF I receptor IGF IR and its major signaling molecule, insulin receptor substrate 1 **[IRS1]** IRS 1 **[IRS1]** .
                  Importantly, IRS 1 **[IRS1]** is translocated to the nucleus in the presence of the JCV T antigen.
                  Nuclear IRS 1 **[IRS1]** was detected in T antigen positive cell lines and in T antigen positive biopsies from patients diagnosed with medulloblastoma.
                  The IRS 1 **[IRS1]** domain responsible for a direct JCV T antigen binding was localized within the N terminal portion of IRS 1 **[IRS1]** molecule and the competition for IRS 1 **[IRS1]** T antigen binding by a dominant negative mutant of IRS 1 **[IRS1]** inhibited growth and survival of JCV T antigen transformed cells in anchorage independent culture condition.

                  - Hepatology, 2002   **Epidermal growth factor induced activation of the insulin like growth factor I receptor **[IGF1R]** in rat hepatocytes.** .
                    Insulin like growth factor I IGF I plays a critical role in the induction of cell cycle progression and survival in many cell types.
                    However, there is minimal IGF I binding to hepatocytes, and a role for IGF I in hepatocyte signaling has not been elucidated.
                    The dynamics of IGF I receptor IGF IR activation were examined in freshly isolated rat hepatocytes.
                    IGF I did not activate the IGF IR.
                    However, des 1 3 IGF I, which weakly binds IGF binding protein 3 IGFBP 3 , induced IGF IR phosphorylation.
                    IGFBP 3 surface coating was identified by confocal immunofluorescence microscopy.
                    In contrast with the inactivity of IGF I, epidermal growth factor EGF induced the tyrosine phosphorylation of the IGF IR in parallel with EGF receptor phosphorylation.
                    Transactivation of the IGF IR by EGF was inhibited by tyrphostin I Ome AG538, a tyrosine kinase inhibitor with high specificity for the IGF IR.
                    Src kinase inhibitors pyrazolopyrimidine PP 1 and PP 2 inhibited transactivation of the IGF IR by EGF.
                    EGF stimulated the tyrosine phosphorylation of Src, and induced its association with the IGF IR.
                    EGF induced phosphorylations of insulin related substrate IRS 1 **[IRS1]**, IRS **[IRS1]** 2, Akt, and p42 44 mitogen activated protein kinases MAPKs were inhibited variably by I Ome AG538.
                    In conclusion, the data show an EGF and Src mediated transactivation pathway for IGF IR activation in hepatocytes, and indicate a role for the IGF IR in hepatocyte intracellular signaling.
                    The findings also show a role for IGFBP 3 in the inhibition of IGF I signaling in hepatocytes.
